# PMA: Protein Microarray Analyser, a user-friendly tool for data processing and normalization

**DOI:** 10.1186/s13104-018-3266-0

**Published:** 2018-02-27

**Authors:** Jessica Da Gama Duarte, Ryan W. Goosen, Peter J. Lawry, Jonathan M. Blackburn

**Affiliations:** 1 Department of Integrative Biomedical Sciences & Institute for Infectious Disease and Molecular Medicine, Faculty of Health Sciences, University of Cape Town, Cape Town, South Africa; 2 Present Address: Tumour Immunology Laboratory, Olivia Newton-John Cancer Research Institute/School of Cancer Medicine, La Trobe University, Level 5, ONJCWC, 145 Studley Road, Heidelberg, VIC 3084 Australia; 3 Present Address: Olivia Newton-John Cancer Research Institute/School of Cancer Medicine, La Trobe University, Level 5, ONJCWC, 145 Studley Road, Heidelberg, VIC 3084 Australia; 4 Present Address: Blackburn Laboratory, N3.03, Wernher & Beit Building North, Institute of Infectious Disease & Molecular Medicine, UCT Faculty of Health Sciences, Observatory, Cape Town, 7925 South Africa

**Keywords:** Protein microarrays, Protein Microarray Analyser, PMA

## Abstract

**Objective:**

Protein microarrays provide a high-throughput platform to measure protein interactions and associated functions, and can aid in the discovery of cancer biomarkers. The resulting protein microarray data can however be subject to systematic bias and noise, thus requiring a robust data processing, normalization and analysis pipeline to ensure high quality and robust results. To date, a comprehensive data processing pipeline is yet to be developed. Furthermore, a lack of analysis consistency is evident amongst different research groups, thereby impeding collaborative data consolidation and comparison. Thus, we sought to develop an accessible data processing tool using methods that are generalizable to the protein microarray field and which can be adapted to individual array layouts with minimal software engineering expertise.

**Results:**

We developed an improved version of a previously developed pipeline of protein microarray data processing and implemented it as an open source software tool, with particular focus on widening its use and applicability. The Protein Microarray Analyser software presented here includes the following tools: (1) neighbourhood background correction, (2) net intensity correction, (3) user-defined noise threshold, (4) user-defined CV threshold amongst replicates and (5) assay controls, (6) composite ‘pin-to-pin’ normalization amongst sub-arrays, and (7) ‘array-to-array’ normalization amongst whole arrays.

**Electronic supplementary material:**

The online version of this article (10.1186/s13104-018-3266-0) contains supplementary material, which is available to authorized users.

## Introduction

Protein microarrays are a high-throughput technology that can measure protein interactions and associated functions, with potential uses in cancer biomarker discovery [1–14]. We have previously developed a custom cancer-specific protein array which measures antigen-specific antibodies present in patient blood [15, 16]. These are quantified using fluorescently-labelled anti-human IgG antibodies and a microarray scanner [17]. However, the resulting data can be subject to systematic bias and noise, and robust data processing and normalization is required to ensure high quality data. DNA microarray tools are generally unsuitable for this purpose given the different assay setup, objectives and statistical assumptions used. Although several protein microarray-specific tools are available [18–25], none of these include a composite suite of methods that we deemed as essential. Furthermore, no consistency is seen amongst research groups, which impedes collaborative data consolidation and comparison. Thus, we sought to develop a generic protein microarray data processing tool that is readily adaptable to any array layout and should thereby provide valuable new insight in the field by enabling collaborative data analysis of new and existing datasets.

## Main text

### ‘CT100 Analyser’

Our group has previously developed ‘CT100 Analyser’ [17], which included the following methods:

#### Neighbourhood background correction

Net intensities are usually calculated by subtracting local background intensities from raw intensities. However, printing, assay and handling artefacts may lead to artificially increased local background intensities, which thereby skew the calculated net intensities. Local background intensities are replaced with median surrounding neighbourhood corrected intensities according to Zhu et al. [24].

#### Corrected net intensity

Net intensities are recalculated by subtracting the corrected background intensity from the raw intensity for each spot.

#### Noise threshold

Non-specific binding can result in background noise that affects antigen-specific net intensity calculations. In addition to experimental methods for reducing noise during array fabrication and assay, a noise threshold can be applied to remove all intensities that are not significantly above background. All spots containing a corrected net intensity of less than two standard deviations of the background are deemed “NOISY” and excluded.

#### Spot filtering

Antigens, positive controls and negative controls are expected to be present at least in triplicate on the array, as a means of assuring that the obtained data is reliable and equally detected across spatially distinct locations. At times, data is not consistent across spot replicas, and the resulting mean net intensity may not be indicative of the true signal. Therefore, after calculating the mean for each set of spot replicas, the coefficient of variation (CV) across these replicas is also calculated. If the obtained CV is above 20% (user-defined), the mean net intensity is deemed “HIGH CV” and excluded.

Saturation occurs when the fluorescent intensity detected surpasses the scanner’s reading capacity, and as a result, this maximum value is reported alongside information regarding the percentage of pixels within the spot of interest that are saturated. To assure that all intensities reported are reliable, all spots that show saturation above 10% are deemed “SATURATED”, and the array flagged for rescanning at a lower PMT gain setting.

#### Array filtering for a selected positive control

Positive controls are essential in all protein microarrays and can be used for the implementation of data filtering and normalization methods. Replicas of these controls should be well distributed across the entire array surface. At times, slide coating, sample loading or printing issues can arise and affect spot homogeneity and size. To investigate whether array printing was up to the expected standard, CVs of a selected positive control are calculated for each array across all replicas. If an array’s CV is above 20%, this array is excluded and flagged as a required repeat.

#### ‘Pin-to-pin’ and ‘array-to-array’ normalization

To enhance assay throughput, multiple replica arrays can be printed across a single slide. However, the usage of multiple pins/nozzles and the replica printing action can lead to slight variations. Moreover, differences in microarray scanner PMT gain settings can complicate data comparison between arrays or datasets. Therefore, data normalization is essential to account for these variations. The above mentioned positive control spatially dispersed replicas are used for this purpose. The functionality of this method requires these controls to be in the defined static location and at three different concentrations. We have implemented a composite normalization method combining quantile normalization and total intensity normalization modules [26–28]. With this method, individual blocks within each array are normalized with respect to each other to minimize any effects of ‘pin-to-pin’ variation, and whole arrays are then normalized with respect to each other to minimize any effects of ‘array-to-array’ variation. This normalization method only uses data points and arrays that have not been flagged or discarded by prior methods.

#### Data consolidation

After processing all raw data files with ‘CT100 Analyser’ an output folder is generated. The final resulting data files are consolidated into a single file, where each column represents a single array, and each row a single antigen/control. Each data point corresponds to the mean net intensity of all valid replicas for each sample. Additionally, flagged and discarded folders are generated containing all problematic data.

### PMA—Protein Microarray Analyser

Extensive use of ‘CT100 Analyser’ highlighted opportunities for further improvement and generalisation, which we have now addressed. The following methods were included:

#### Slide scanning using the automatic gain control (AGC) mode

Fluorescent microarray scanners have an AGC mode in addition to user-defined PMT gain settings. The former ensures that no saturating signals are detected throughout the array thereby excluding the need to flag saturated spots and rescan slides. PMA thus now allows for scanning arrays using the AGC mode. However, it is important to note that when using this setting, subsequent data normalization is critical.

#### A user-defined antigen layout and list (.gal file)

After scanning, users create or input a.gal file according to the specific array layout and antigen list to enable adequate data extraction. The functionality of ‘CT100 Analyser’ was restricted to a fixed.gal file and any modifications to the array layout required adaptation of this tool accordingly. Since different arrays have different content and layouts, we therefore sought to make the software dynamic and applicable to any antigen layout, while maintaining the same use of positive and negative controls. PMA now enables processing of raw data extracted with a user-defined.gal file, as long as this file is included in the program folder. Currently implemented methods in PMA require the inclusion of specific, statically-defined positive and negative controls to ensure their correct functionality in subsequent quality control, slide orientation and signal normalization steps. It is therefore important to note this when adapting this source code to an alternative array layout.

Additional positive controls (e.g. anti-human IgG and human IgG spots, to confirm respectively the addition of patient serum/plasma and detection antibody) and negative controls (e.g. buffer-only and tag-only, to determine any non-specific immunochemical interactions) are also allowed for in PMA.

#### A user-defined adjustable noise threshold

Despite best efforts, the amount of noise detected on protein arrays can be variable across different assay runs. As such, the noise threshold is now user-defined as *n* standard deviations of the background.

#### Array filtering for each positive control

The ‘CT100 Analyser’ previously used positive controls at three distinct concentrations for array filtering and normalization purposes, conducting CV calculations and discarding flagged arrays using the user-defined concentration of positive controls. However, it was previously necessary to test this function across all three concentrations of positive controls, requiring the user to conduct three separate analyses, each generating a different final consolidated data file. The improved PMA software applies this method using all three different concentrations of positive controls, but proceeds with the processing pipeline using the user-selected control. This ensures that the user is informed of which control is best for this purpose, and also highlights any potential printing concerns that may not have been apparent previously.

#### An improved mean net intensity calculation

After all methods have been applied, replica spots for each antigen or control are averaged, and a mean net intensity is reported. However, a mean isn’t reported in two instances—when one of the replicas is “NOISY” (intensity below the user-defined noise threshold) or when the mean is “HIGH CV” (CV of the replicas above the user-defined percentage). This mean calculation has now been improved to avoid losing valid data when only one of the three replicas is problematic. Specifically, the mean is calculated when two or three of the replicas are available and distributed with low-variance (outlier replicas excluded). As a result, skewed means are avoided and fewer data points are unnecessarily discarded from the analysis.

#### A user-friendly output folder

The ‘CT100 Analyser’ output contained an excessive amount of information that was not user-friendly. We have improved the content of this output folder by only including the processed files that are relevant for downstream data analysis. These include the final consolidated replica and averaged data for all arrays after application of all methods and the list of discarded arrays that require repetition. Additionally, this folder is dated and timed and includes a record of the used settings. Verbose processing output may also be viewed when executing the PMA program via the command line when additional information is required.

### Implementation

PMA is a desktop-based offline Java tool that supports.txt file formats, which are the standard protein microarray image acquisition and analysis software output.

#### Running the software

**Fig. 1:**
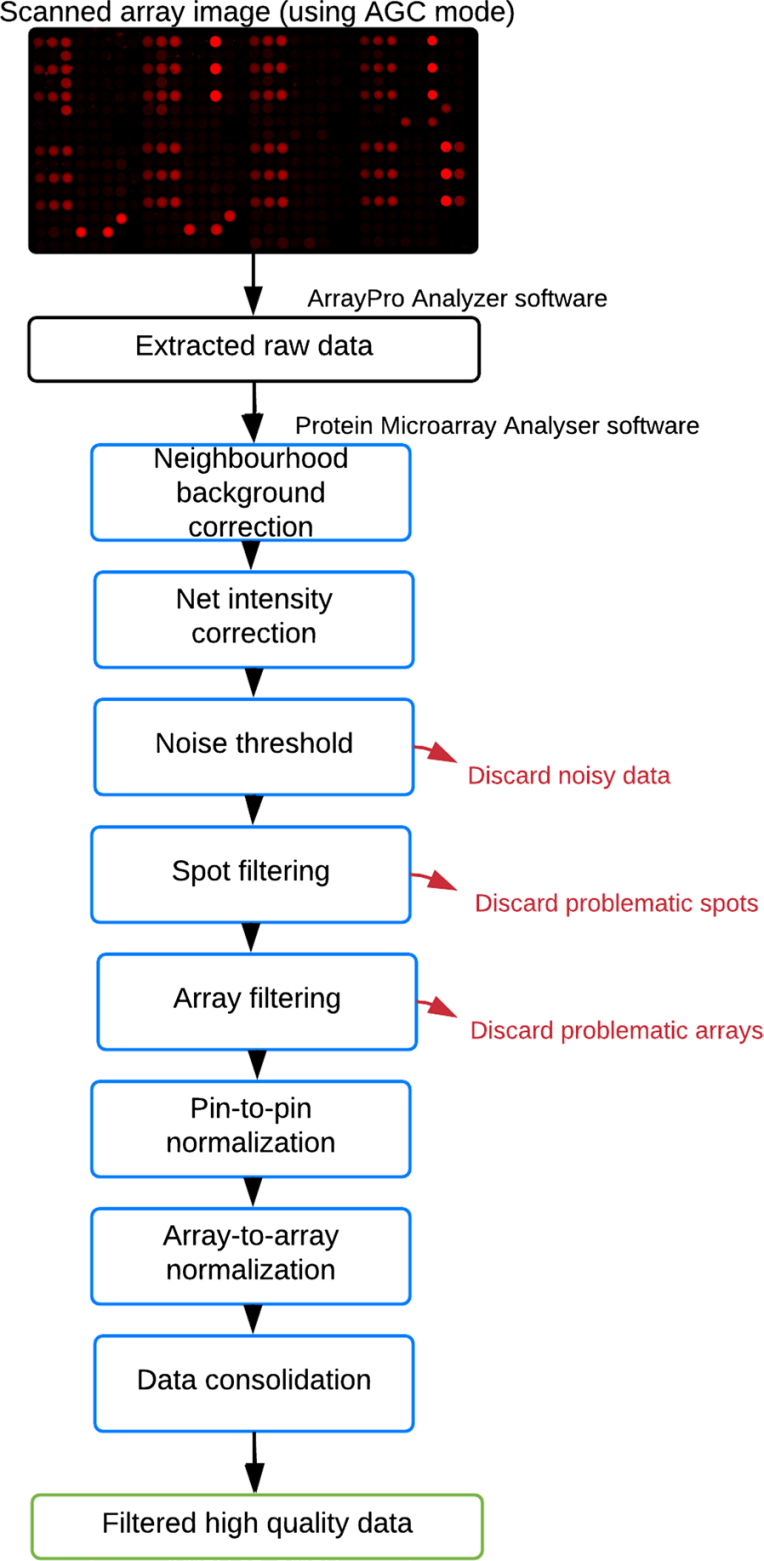
Schematic of the Protein Microarray Analyser data processing and normalization pipeline. Extracted raw data is corrected and filtered to remove or flag problematic data and obtain high quality results that can then be used across a multitude of appropriate data analysis tools

PMA includes: (1) neighbourhood background correction, (2) net intensity correction, (3) user-defined noise threshold, (4) user-defined replicate and (5) control CV threshold, (6) composite ‘pin-to-pin’ and (7) ‘array-to-array’ normalization (Fig. 1).

**Fig. 2:**
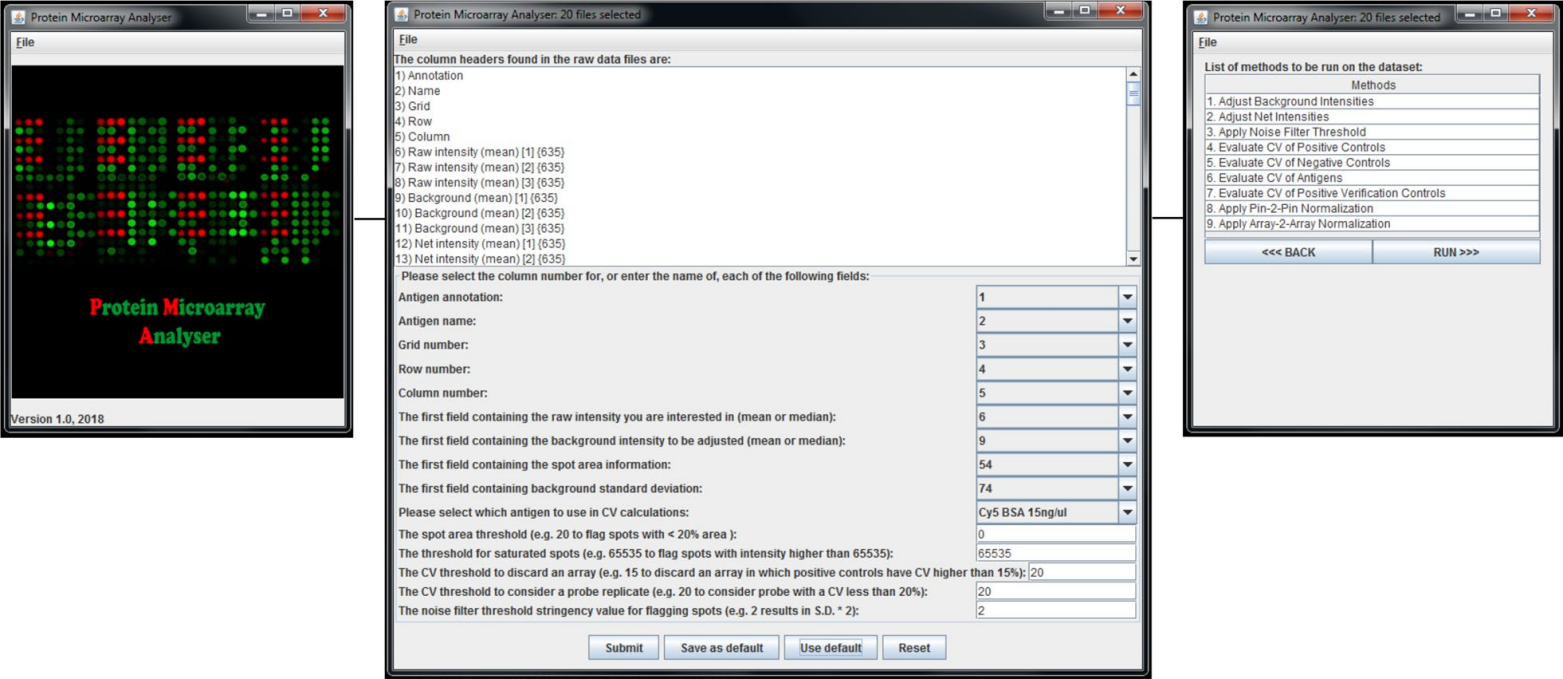
Protein Microarray Analyser software interface. This user-friendly interface allows the user to select raw data files, select default or custom settings, and lists the methods to be run on the dataset

The software can be executed via the GUI (.jar file, Fig. 2) or command line (java -jar ProteinMicroarrayAnalyser.jar > output.txt). Individual.txt raw data files for each array assayed should be placed in a single folder, and this folder should be selected by the user as a new dataset (select file—new dataset—select folder containing raw data.txt files). The user is then required to either input personalized settings or to select the default setting option. The default settings are based on previously published array layouts and as such should be reviewed and adjusted accordingly. After submitting the user-defined settings, the next interface lists the methods to be run on the dataset, after which the analysis is initiated and a results folder is generated.

#### After the software run

The results folder is automatically named with the date and time of the run, and includes two consolidated files. These tab-delimited files include the final RFU values across arrays with all replicas (ProteinMicroarrayAnalyser.AllAntigenReplicateValues.txt) or single averages (ProteinMicroarrayAnalyser.consolidated.txt).

Four separate tab-delimited CV evaluation files may also be included in this results folder. These are generated using low (listOfArraysToDiscard.low.txt), medium (listOfArraysToDiscard.med.txt), high (listOfArraysToDiscard.high.txt) or the selected (listOfArraysToDiscard.overall.txt) positive controls. These include lists of arrays that have failed the user-defined CV threshold with that particular control, and thus require repetition.

The debug output enables users to obtain specific details of each array processed, if required. Manual visualization of all scanned arrays is recommended and should assess spot-to-spot variation, spot homogeneity, background variation, signal-to-noise ratio and saturated pixels [17].

#### Data analysis

The file used for analysis is the average consolidated file, and can be viewed using a spreadsheet or text editor. Rows correspond to antigens, columns to arrays, and array names to raw file location. Data points consist of RFU values, or the terms “NOISY” or “HIGH CV”. “NOISY” data should be set to zero and “HIGH CV” antigens excluded from analysis. A worked example demonstrates the implementation of this tool in more detail (see Additional files 1, 2).

There are a large number of readily available downstream data analysis tools that can be used on protein microarray data, and as such we did not include these. Tool selection should depend on the analysed cohort and research question.

### Limitations

PMA functionality requires specific positive and negative controls in a defined static location, does not permit individual method usage or include subsequent statistical data analysis methods.

## Additional files

**Additional file 1.** A worked example of Protein Microarray Analyser. This example includes a step-by-step description using a real dataset generated with our custom protein array.

**Additional file 2.** Protein Microarray Analyser source code archive. This archive includes the Protein Microarray Analyser source code, the executable jar file, the default settings, and the necessary (raw data folder, .gal file) and generated (results folder) files for a worked example.

## Abbreviations

PMA: Protein Microarray Analyser
AGC: automatic gain control
CV: coefficient of variation
PMT: photomultiplier tube
RFU: relative fluorescence units
SD: standard deviation
