# Supplementary material for: PMA: Protein Microarray Analyser, a user-friendly tool for data processing and normalization
Source: BMC Res Notes. 2018 Feb 27;11:156. doi: 10.1186/s13104-018-3266-0 (PMC5828362; doi:10.1186/s13104-018-3266-0)
Supplement: Supplementary file 1 — Additional file 1. A worked example of Protein Microarray Analyser. This example includes a step-by-step description using a real dataset generated with our custom protein array. [file 13104_2018_3266_MOESM1_ESM.docx]

***Additional Material***

**PMA: Protein Microarray Analyser - a user-friendly tool for data processing and normalization**

Jessica Da Gama Duarte*, Ryan W. Goosen, Peter J. Lawry and Jonathan M. Blackburn

*corresponding author

Additional file 1: A worked example of Protein Microarray Analyser.

We applied PMA to a real raw dataset generated using our custom cancer-specific protein array which measures antigen-specific antibodies present in patient blood. All used input files and generated output files are included in supplementary additional file 2. This dataset included 20 raw data files (RAW DATA folder), corresponding to 5 patients (labelled 1 to 5) at 4 different time points (labelled A to D). The resulting data files (results_2018-02-13_162000 folder) were generated using our recommended default settings (settings_2018-02-13_161958.dat file).

Array filtering using the high concentration of positive controls identified 3 problematic arrays in the listOfArraysToDiscard.overall.txt file. These were arrays 3A, 3B and 3C, which were automatically excluded prior to the application of the normalization methods.

The resulting consolidated averages file is detailed in the ProteinMicroarrayAnalyser.consolidated.txt file. This is the file used for analysis, and was viewed and edited using Microsoft Excel (ProteinMicroarrayAnalyser.consolidated.xlsx). Rows correspond to each 123 antigens or controls, columns to each accepted array (1A to 1D, 2A to 2D, 3D, 4A to 4D, 5A to 5D), and array names to the raw file location. Data points consist of a RFU value, or the terms “NOISY” or “HIGH CV”. In the “With zeros” sheet all “NOISY” and “HIGH CV” data points were set to zero. All instances of “HIGH CV” were restricted to controls, and as such no antigens were excluded from analysis. In the “Patient data” sheet, all arrays except 3D (time points A, B and C missing due to exclusion by the array filtering method) and resulting antibody data were included across all antigens.

For data visualization purposes, positive and negative controls across all arrays were graphed in the “Controls” sheet, and patient data across patients 1, 2, 4 and 5 were graphed in separate sheets “1”, “2”, “4” and “5”. Distinct antibody profiles were seen for each patient, with high antigen-specific average RFU values often seen consistently across multiple time points for each patient, as expected. Downstream statistical data analysis tools can be subsequently applied to this processed and normalized data.
